# Supplementary material for: Identification of photoperiod-induced specific miRNAs in the adrenal glands of Sunite sheep (Ovis aries)
Source: Front Vet Sci. 2022 Jul 22;9:888207. doi: 10.3389/fvets.2022.888207 (PMC9354845; doi:10.3389/fvets.2022.888207)
Supplement: Supplementary file 1 [file Data_Sheet_1.ZIP › Supplementary file/miRDeep2 file.zip!/miRDeep2 file/Know_Structure/SP42A3/oar-let-7c.pdf]

miRBase precursor : oar-let-7c
Total read count : 110309
oar-let-7c read count : 110228
remaining reads : 81

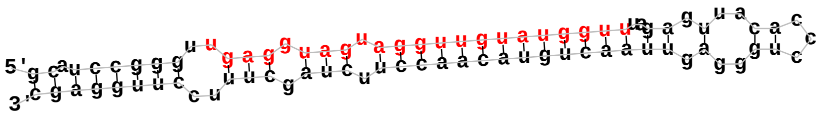

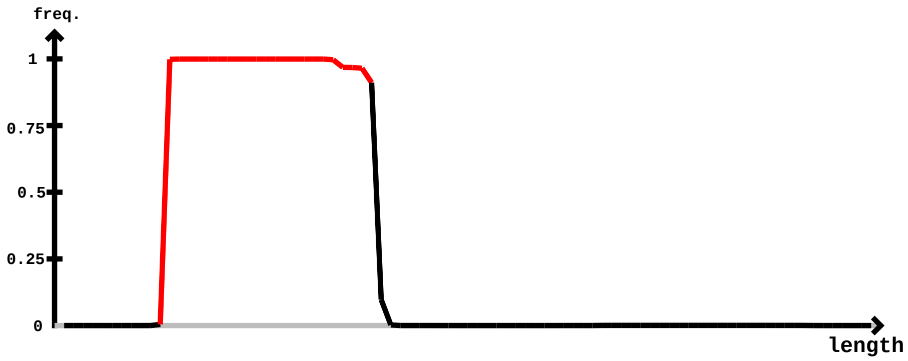

oar-let-7c

5'- gcauccggguugagguaguagguuguaugguuuagaguuacacccugggaguuaacuguacaaccuucuagcuuuccuuggagc -3'	exp		
((.((((((..(((.(((.(((((((((((((..((.(..((...))..).))))))))))))))).))).)))..))))))))	reads	mm	sample
........guugagguaguagguuguauggu...	3	0	seq
........guugagguaguagguuguaugguu..	2	0	seq
........guugagguaguagguuguaugguuu...	1	0	seq
.........uugagguaguagguuguau..	3	0	seq
.........uugagguaguagguuguaugg..	3	0	seq
.........uugagguaguagguuguauggu...	102	0	seq
.........uugagguaguagguuguauAgu...	2	1	seq
.........uugagguaguagguuguaugguu..	332	0	seq
.........uugagguaguagguuguauAguu..	7	1	seq
.........uugagguaguagguuguaugguuu...	25	0	seq
..........ugagguaguagguuUua...	2	1	seq
..........ugagguaguaAguugua...	13	1	seq
..........ugagguaguagguugua...	235	0	seq
..........ugagguagGagguugua...	33	1	seq
..........ugaAguaguagguugua...	1	1	seq
..........ugagguUguagguugua...	1	1	seq
..........ugagguaguagguuguau..	2830	0	seq
..........ugagguaguagguuUuau..	13	1	seq
..........ugagguaguaAguuguau..	16	1	seq
..........ugagguUguagguuguau..	1	1	seq
..........ugaAguaguagguuguau..	16	1	seq
..........ugagguagGagguuguau..	262	1	seq
..........ugagguaguagguuguaug...	75	0	seq
..........ugagguaguagguuguaugg..	293	0	seq
..........ugagguaguagguuguauggu...	5833	0	seq
..........ugagguaguagguuguaugguu..	89345	0	seq
..........ugagguaguagguuguaugguuu...	10415	0	seq
..........ugagguaguagguuguauAguuua..	189	1	seq
..........ugagguaguagguuguaugguuua..	73	0	seq
..........ugagguaguagguuguaugguuuag...	1	0	seq
..........ugagguaguagguuguaugguuuagagu..	4	0	seq
..........ugagguaguagguuguaugguuuagaguuac...	2	0	seq
...........gagguagGagguuguau..	1	1	seq
...........gagguaguagguuguau..	1	0	seq
...........gagguaguagguuguaugg..	1	0	seq

oar-let-7c

```
gcauccggguugagguaguagguuguaugguuuagaguuacacccugggaguuaacuguacaaccuucuagcuuuccuuggagc
...........gagguaguagguuguauggu.....................................................    2    0    seq
...........gagguaguagguuguaugguu....................................................    82   0    seq
...........gagguaguagguuguaugguuu...................................................    11   0    seq
............agguaguagguuguaugguu....................................................    1    0    seq
............agguaguagguuguaugguuu...................................................    1    0    seq
.............gguaguagguuguaugguu....................................................    1    0    seq
................................uagaguuacacccugggaguu...............................    1    0    seq
.................................................aguuaacuguacaaccuucuagcu...........    4    0    seq
...................................................uuaacuguacaaccuucuagcuuu.........    1    0    seq
....................................................uaacuguacaaccuucuagcu...........    1    0    seq
.....................................................aacuguacaaccuucuagcu...........    1    0    seq
.....................................................aacuguacaaccuucuagcuuu.........    1    0    seq
.....................................................aacuguacaaccuucuagcuuuc........    1    0    seq
.......................................................cuguacaaccuucuagcuuu.........    2    0    seq
.......................................................cuguacaaccuucuagcuuuc........    9    0    seq
.......................................................cuguacaaccuucuagcuuucc.......    38   0    seq
.......................................................cuguacaaccuucuagcuuuccu......    13   0    seq
........................................................uguacaaccuucuagcuuuccu......    3    0    seq
```
